# *N*′-[(*E*)-2-Chloro­benzyl­idene]thio­phene-2-carbohydrazide

**DOI:** 10.1107/S1600536813020850

**Published:** 2013-08-17

**Authors:** Ismail Warad, Salim F. Haddad, Mousa Al-Noaimi, Belkheir Hammouti, Taibi Ben Hadda

**Affiliations:** a Department of Chemistry, An-Najah National University, Nablus, Palestinian Territories; b Department of Chemistry, The University of Jordan, Amman 11942, Jordan; c Department of Chemistry, Hashemite University, PO Box 150459, Zarqa 13115, Jordan; d LCAE-URAC18, Faculté des Sciences, Université Mohammed Ier, Oujda-60000, Morocco; e Laboratoire LCM, Faculté des Sciences, Université Mohammed Ier, Oujda-60000, Morocco

## Abstract

There are two independent mol­ecules in the asymmetric unit of the title compound, C_12_H_9_ClN_2_OS, a Schiff base derived from hydrazide, in which the dihedral angles between the thio­phene and benzene rings are 3.6 (3) and 7.3 (3)°. In the crystal, the two independent mol­ecules are arranged about an approximate non-crystallographic inversion center and are connected by two N—H⋯O hydrogen bonds. Weak C—H⋯Cl contacts are also present. Conversely, there are neither significant aromatic stacking inter­actions nor contacts involving S atoms.

## Related literature

For applications of Schiff bases, see: Cimerman *et al.* (1997 ▶); Ren *et al.* (2002 ▶). For related structures, see: Warad *et al.* (2009 ▶); Jiang (2010 ▶, 2011 ▶); Li & Jian (2010 ▶); Li & Meng (2010 ▶).
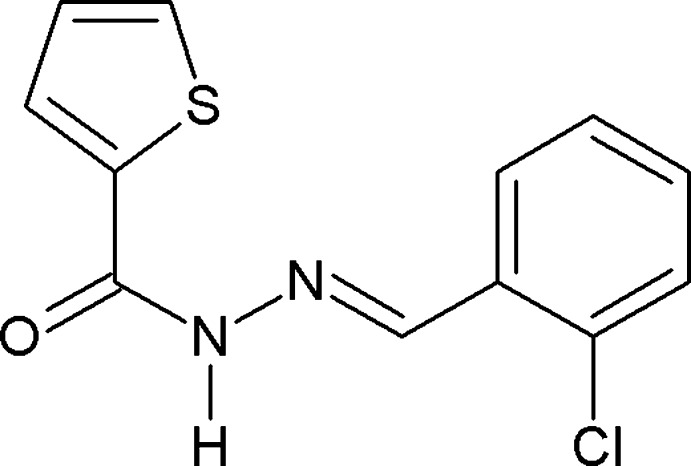


## Experimental

### 

#### Crystal data

C_12_H_9_ClN_2_OS
*M* *_r_* = 264.72
Orthorhombic, 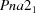

*a* = 24.9465 (15) Å
*b* = 4.3709 (3) Å
*c* = 21.8091 (13) Å
*V* = 2378.0 (2) Å^3^
*Z* = 8
Mo *K*α radiation
μ = 0.48 mm^−1^
*T* = 293 K
0.30 × 0.20 × 0.10 mm

#### Data collection

Agilent Xcalibur Eos diffractometer
Absorption correction: multi-scan (*CrysAlis PRO*; Agilent, 2011 ▶) *T*_min_ = 0.870, *T*_max_ = 0.954
9965 measured reflections
3845 independent reflections
3341 reflections with *I* > 2σ(*I*)
*R*_int_ = 0.025

#### Refinement

*R*[*F* ^2^ > 2σ(*F* ^2^)] = 0.044
*wR*(*F* ^2^) = 0.113
*S* = 1.08
3845 reflections
316 parameters
1 restraint
H atoms treated by a mixture of independent and constrained refinement
Δρ_max_ = 0.32 e Å^−3^
Δρ_min_ = −0.32 e Å^−3^

### 

Data collection: *CrysAlis PRO* (Agilent, 2011 ▶); cell refinement: *CrysAlis PRO*; data reduction: *CrysAlis PRO*; program(s) used to solve structure: *SHELXS97* (Sheldrick, 2008 ▶); program(s) used to refine structure: *SHELXL97* (Sheldrick, 2008 ▶); molecular graphics: *ORTEPIII* (Burnett & Johnson, 1996 ▶); software used to prepare material for publication: *SHELXTL* (Sheldrick, 2008 ▶).

## Supplementary Material

Crystal structure: contains datablock(s) I, New_Global_Publ_Block. DOI: 10.1107/S1600536813020850/bh2481sup1.cif

Structure factors: contains datablock(s) I. DOI: 10.1107/S1600536813020850/bh2481Isup2.hkl

Supplementary material file. DOI: 10.1107/S1600536813020850/bh2481Isup3.cml

Additional supplementary materials:  crystallographic information; 3D view; checkCIF report

## Figures and Tables

**Table 1:** Hydrogen-bond geometry (Å, °)

*D*—H⋯*A*	*D*—H	H⋯*A*	*D*⋯*A*	*D*—H⋯*A*
N3—H3*A*⋯O1^i^	0.84 (4)	2.02 (5)	2.856 (4)	175 (4)
N1—H1*A*⋯O2^ii^	0.77 (4)	2.09 (4)	2.845 (5)	166 (5)
C10—H10*A*⋯Cl2^iii^	0.93	2.93	3.758 (5)	149

Symmetry codes: (i) 
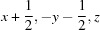
; (ii) 
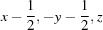
; (iii) 
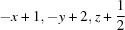
.

**Fig. 1.**
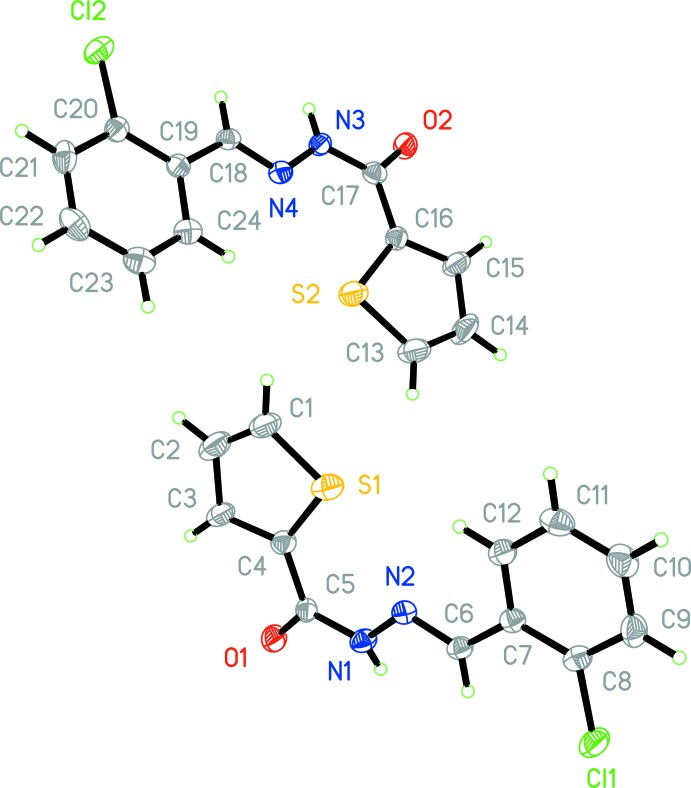
ORTEP drawing showing molecular conformation for the title compound. Displacement ellipsoids are drawn at the 30% probability level. Hydrogen atoms are represented as small spheres of arbitrary radii.

**Fig. 2.**
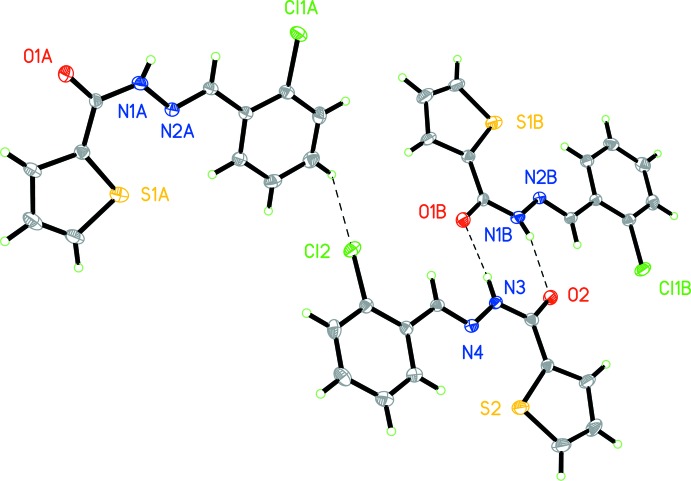
ORTEP drawing with 20% displacement ellipsoids showing N—H···O and C—H···Cl hydrogen bonding.

## supplementary crystallographic information

### 1. Comment

Schiff bases derivatives have attracted much attention due to their pharmacological activity (Ren *et al.*, 2002). They are attractive from several points of view, such as the possibility of analytical applications (Cimerman *et al.*, 1997). As part of our search for new Schiff base compounds as ligand (Warad *et al.*, 2009), we synthesized the title compound, and describe its structure here. The title compound crystallizes with two independent molecules in the asymmetric unit, related by non-crystallographic inversion. The dihedral angles between the aromatic rings in the two independent molecules are surprisingly different, 3.6 (3)° for S1-molecule and 7.3 (3)° for S2-molecule (Fig. 1). In the crystal lattice two rather strong N—H···O intermolecular hydrogen bonds hold the two independent molecules together. One CH group of the benzene ring is also hydrogen bonded to the Cl atom of the chlorophenyl ring of a symmetry-related independent molecule (Fig. 2). There are no intermolecular contacts to sulfur of importance. Bond lengths and angles are comparable to those in related compounds (Warad *et al.*, 2009; Jiang, 2010, 2011; Li & Jian, 2010; Li & Meng, 2010).

### 2. Experimental

A mixture of thiophene-2-carbohydrazide (0.05 mol), and 2-chlorobenzaldehyde (0.05 mol) was stirred in refluxing ethanol (20 ml) for 3 h to afford the title compound (0.092 mol, yield 92%). Single crystals suitable for X-ray measurements were obtained by re-crystallization of the title compound from ethanol, at room temperature. A set of 265 frames with an exposure time of 44.4 s per frame was collected.

### 3. Refinement

All non-H atoms were refined anisotropically. H atoms attached to C atoms were positioned geometrically, with C—H = 0.93 Å, and constrained to ride on their parent atoms, with *U*_iso_(H) = 1.2*U*_eq_(carrier C). H atoms bonded to N1 and N3 were located in a difference map, and refined freely.

### Crystal data

**Table:**

C_12_H_9_ClN_2_OS	*F*(000) = 1088
*M**_r_* = 264.72	*D*_x_ = 1.479 Mg m^−^^3^
Orthorhombic, *P**n**a*2_1_	Mo *K*α radiation, λ = 0.71073 Å
Hall symbol: P 2c -2n	Cell parameters from 3090 reflections
*a* = 24.9465 (15) Å	θ = 2.9–29.2°
*b* = 4.3709 (3) Å	µ = 0.48 mm^−^^1^
*c* = 21.8091 (13) Å	*T* = 293 K
*V* = 2378.0 (2) Å^3^	Parallelpiped, colourless
*Z* = 8	0.30 × 0.20 × 0.10 mm

### Data collection

**Table:**

Agilent Xcalibur Eos diffractometer	3845 independent reflections
Radiation source: Enhance (Mo) X-ray Source	3341 reflections with *I* > 2σ(*I*)
Graphite monochromator	*R*_int_ = 0.025
Detector resolution: 16.0534 pixels mm^-1^	θ_max_ = 25.0°, θ_min_ = 3.2°
ω scans	*h* = −29→29
Absorption correction: multi-scan (*CrysAlis PRO*; Agilent, 2011)	*k* = −5→4
*T*_min_ = 0.870, *T*_max_ = 0.954	*l* = −25→21
9965 measured reflections	

### Refinement

**Table:**

Refinement on *F*^2^	Primary atom site location: structure-invariant direct methods
Least-squares matrix: full	Secondary atom site location: difference Fourier map
*R*[*F*^2^ > 2σ(*F*^2^)] = 0.044	Hydrogen site location: inferred from neighbouring sites
*wR*(*F*^2^) = 0.113	H atoms treated by a mixture of independent and constrained refinement
*S* = 1.08	*w* = 1/[σ^2^(*F*_o_^2^) + (0.0575*P*)^2^ + 0.3988*P*] where *P* = (*F*_o_^2^ + 2*F*_c_^2^)/3
3845 reflections	(Δ/σ)_max_ < 0.001
316 parameters	Δρ_max_ = 0.32 e Å^−^^3^
1 restraint	Δρ_min_ = −0.32 e Å^−^^3^
0 constraints	

### Fractional atomic coordinates and isotropic or equivalent isotropic displacement parameters (Å^2^)

**Table:**

	*x*	*y*	*z*	*U*_iso_*/*U*_eq_	
S1	0.30063 (4)	0.4329 (3)	0.72298 (6)	0.0619 (3)	
O1	0.17505 (11)	−0.0648 (6)	0.67301 (13)	0.0509 (7)	
N1	0.17938 (14)	0.2450 (8)	0.7547 (2)	0.0414 (9)	
H1A	0.1555 (17)	0.166 (10)	0.770 (2)	0.046 (13)*	
N2	0.20518 (12)	0.4548 (7)	0.78995 (15)	0.0363 (7)	
Cl1	0.11381 (5)	0.7410 (3)	0.94945 (7)	0.0663 (4)	
C1	0.34789 (17)	0.3764 (12)	0.6679 (2)	0.0598 (12)	
H1	0.3823	0.4576	0.6701	0.072*	
C2	0.33115 (19)	0.2071 (12)	0.6219 (3)	0.0684 (16)	
H2B	0.3532	0.1601	0.5889	0.082*	
C3	0.27627 (15)	0.0975 (10)	0.6257 (2)	0.0502 (11)	
H3	0.2582	−0.0210	0.5969	0.060*	
C4	0.25557 (16)	0.2105 (8)	0.6832 (2)	0.0412 (11)	
C5	0.20111 (14)	0.1229 (9)	0.70353 (18)	0.0372 (9)	
C6	0.17984 (14)	0.5461 (8)	0.83769 (19)	0.0376 (9)	
H6A	0.1454	0.4745	0.8454	0.045*	
C7	0.20468 (14)	0.7612 (8)	0.8799 (2)	0.0357 (10)	
C8	0.17872 (15)	0.8624 (8)	0.93273 (19)	0.0427 (9)	
C9	0.20295 (19)	1.0600 (10)	0.9746 (2)	0.0527 (11)	
H9A	0.1849	1.1230	1.0098	0.063*	
C10	0.25406 (18)	1.1590 (11)	0.9628 (3)	0.0580 (13)	
H10A	0.2707	1.2914	0.9902	0.070*	
C11	0.28077 (17)	1.0671 (9)	0.9117 (2)	0.0551 (11)	
H11A	0.3156	1.1352	0.9048	0.066*	
C12	0.25639 (14)	0.8721 (9)	0.8698 (2)	0.0451 (10)	
H12A	0.2748	0.8147	0.8345	0.054*	
S2	0.45222 (4)	−0.0291 (3)	0.74612 (7)	0.0598 (3)	
O2	0.57997 (11)	−0.5028 (7)	0.79635 (13)	0.0506 (7)	
N3	0.57520 (13)	−0.1945 (7)	0.71524 (18)	0.0367 (8)	
H3A	0.6055 (17)	−0.260 (9)	0.705 (2)	0.050 (13)*	
N4	0.54883 (11)	0.0161 (7)	0.67961 (15)	0.0365 (7)	
Cl2	0.63843 (4)	0.3111 (3)	0.52014 (6)	0.0597 (3)	
C13	0.40651 (17)	−0.0776 (12)	0.8022 (3)	0.0656 (14)	
H13	0.3718	−0.0001	0.8000	0.079*	
C14	0.4247 (2)	−0.2383 (13)	0.8504 (3)	0.0694 (17)	
H14A	0.4040	−0.2836	0.8846	0.083*	
C15	0.47921 (17)	−0.3327 (11)	0.8438 (2)	0.0565 (12)	
H15	0.4986	−0.4426	0.8728	0.068*	
C16	0.49924 (15)	−0.2344 (8)	0.7868 (2)	0.0384 (10)	
C17	0.55391 (15)	−0.3165 (9)	0.7665 (2)	0.0401 (10)	
C18	0.57349 (13)	0.1124 (8)	0.63301 (19)	0.0356 (8)	
H18A	0.6082	0.0442	0.6253	0.043*	
C19	0.54850 (14)	0.3293 (8)	0.5909 (2)	0.0352 (9)	
C20	0.57460 (14)	0.4371 (8)	0.53871 (18)	0.0390 (9)	
C21	0.54954 (18)	0.6404 (9)	0.4982 (2)	0.0497 (10)	
H21A	0.5678	0.7094	0.4636	0.060*	
C22	0.49814 (19)	0.7381 (9)	0.5093 (3)	0.0558 (14)	
H22A	0.4811	0.8710	0.4823	0.067*	
C23	0.47194 (17)	0.6338 (10)	0.5620 (2)	0.0523 (11)	
H23A	0.4373	0.7006	0.5705	0.063*	
C24	0.49638 (16)	0.4368 (9)	0.6010 (2)	0.0441 (10)	
H24A	0.4779	0.3706	0.6355	0.053*	

### Atomic displacement parameters (Å^2^)

**Table:**

	*U*^11^	*U*^22^	*U*^33^	*U*^12^	*U*^13^	*U*^23^
S1	0.0423 (5)	0.0769 (8)	0.0665 (8)	−0.0039 (6)	0.0064 (5)	−0.0024 (7)
O1	0.0488 (15)	0.0610 (17)	0.0427 (17)	−0.0089 (14)	0.0008 (13)	−0.0127 (15)
N1	0.0348 (18)	0.0425 (19)	0.047 (3)	−0.0051 (15)	0.0052 (18)	−0.0039 (16)
N2	0.0339 (15)	0.0370 (17)	0.0382 (19)	−0.0004 (13)	0.0010 (14)	−0.0001 (15)
Cl1	0.0465 (6)	0.0949 (9)	0.0576 (9)	−0.0116 (6)	0.0170 (6)	−0.0179 (7)
C1	0.039 (2)	0.073 (3)	0.067 (4)	0.004 (2)	0.014 (2)	0.008 (3)
C2	0.056 (3)	0.080 (4)	0.070 (5)	0.017 (3)	0.026 (3)	0.006 (3)
C3	0.038 (2)	0.058 (2)	0.055 (3)	0.0039 (18)	0.014 (2)	0.003 (2)
C4	0.038 (2)	0.043 (2)	0.043 (3)	0.0051 (16)	0.0018 (19)	0.0027 (19)
C5	0.0393 (19)	0.042 (2)	0.031 (2)	0.0044 (17)	0.0010 (16)	0.0014 (19)
C6	0.0319 (17)	0.039 (2)	0.042 (2)	−0.0008 (16)	0.0034 (16)	0.0036 (18)
C7	0.035 (2)	0.0319 (19)	0.040 (3)	0.0033 (14)	0.0008 (17)	0.0033 (17)
C8	0.0385 (19)	0.042 (2)	0.048 (3)	0.0041 (17)	0.0052 (17)	0.001 (2)
C9	0.064 (3)	0.052 (3)	0.042 (3)	0.003 (2)	−0.001 (2)	−0.011 (2)
C10	0.061 (3)	0.053 (3)	0.059 (4)	−0.007 (2)	−0.010 (3)	−0.015 (3)
C11	0.041 (2)	0.058 (3)	0.067 (3)	−0.010 (2)	−0.008 (2)	0.006 (3)
C12	0.040 (2)	0.049 (2)	0.046 (3)	−0.0008 (18)	0.0025 (18)	−0.003 (2)
S2	0.0419 (5)	0.0785 (8)	0.0590 (7)	0.0063 (5)	0.0047 (5)	0.0152 (7)
O2	0.0478 (15)	0.0633 (17)	0.0407 (17)	0.0165 (13)	0.0013 (14)	0.0141 (15)
N3	0.0345 (17)	0.0403 (17)	0.035 (2)	0.0015 (14)	0.0038 (16)	0.0036 (16)
N4	0.0341 (15)	0.0363 (16)	0.039 (2)	0.0009 (13)	0.0017 (14)	0.0008 (15)
Cl2	0.0472 (6)	0.0745 (7)	0.0574 (9)	−0.0028 (5)	0.0165 (6)	0.0058 (7)
C13	0.040 (2)	0.081 (3)	0.076 (4)	0.006 (2)	0.009 (2)	0.019 (3)
C14	0.067 (3)	0.088 (4)	0.053 (4)	0.006 (3)	0.026 (3)	0.024 (3)
C15	0.042 (2)	0.072 (3)	0.056 (3)	0.008 (2)	0.015 (2)	0.006 (3)
C16	0.041 (2)	0.039 (2)	0.035 (3)	−0.0035 (16)	0.0003 (19)	0.0010 (17)
C17	0.0366 (19)	0.040 (2)	0.044 (3)	−0.0036 (17)	−0.0027 (17)	−0.0040 (19)
C18	0.0311 (16)	0.0359 (19)	0.040 (2)	−0.0023 (15)	0.0006 (16)	−0.0043 (18)
C19	0.037 (2)	0.0332 (19)	0.035 (3)	−0.0094 (16)	−0.0012 (17)	−0.0077 (17)
C20	0.0425 (19)	0.038 (2)	0.037 (2)	−0.0054 (16)	0.0015 (16)	−0.0065 (18)
C21	0.070 (3)	0.044 (2)	0.036 (2)	−0.010 (2)	−0.002 (2)	0.005 (2)
C22	0.066 (3)	0.044 (2)	0.058 (4)	0.005 (2)	−0.020 (3)	0.003 (2)
C23	0.044 (2)	0.050 (2)	0.062 (3)	0.004 (2)	−0.005 (2)	−0.003 (2)
C24	0.041 (2)	0.041 (2)	0.050 (3)	−0.0003 (17)	0.0006 (17)	−0.003 (2)

### Geometric parameters (Å, º)

**Table:**

S1—C1	1.701 (5)	S2—C13	1.686 (5)
S1—C4	1.720 (5)	S2—C16	1.723 (4)
O1—C5	1.240 (4)	O2—C17	1.229 (5)
N1—C5	1.350 (5)	N3—C17	1.347 (6)
N1—N2	1.359 (5)	N3—N4	1.373 (4)
N1—H1A	0.77 (4)	N3—H3A	0.84 (4)
N2—C6	1.282 (5)	N4—C18	1.260 (5)
Cl1—C8	1.743 (4)	Cl2—C20	1.733 (4)
C1—C2	1.314 (8)	C13—C14	1.342 (7)
C1—H1	0.9300	C13—H13	0.9300
C2—C3	1.453 (6)	C14—C15	1.427 (6)
C2—H2B	0.9300	C14—H14A	0.9300
C3—C4	1.445 (7)	C15—C16	1.407 (7)
C3—H3	0.9300	C15—H15	0.9300
C4—C5	1.479 (5)	C16—C17	1.478 (5)
C6—C7	1.454 (6)	C18—C19	1.459 (6)
C6—H6A	0.9300	C18—H18A	0.9300
C7—C8	1.393 (6)	C19—C20	1.394 (6)
C7—C12	1.396 (5)	C19—C24	1.400 (5)
C8—C9	1.395 (6)	C20—C21	1.401 (6)
C9—C10	1.371 (6)	C21—C22	1.373 (6)
C9—H9A	0.9300	C21—H21A	0.9300
C10—C11	1.358 (7)	C22—C23	1.399 (7)
C10—H10A	0.9300	C22—H22A	0.9300
C11—C12	1.390 (6)	C23—C24	1.354 (6)
C11—H11A	0.9300	C23—H23A	0.9300
C12—H12A	0.9300	C24—H24A	0.9300
			
C1—S1—C4	90.9 (3)	C13—S2—C16	91.2 (2)
C5—N1—N2	123.0 (4)	C17—N3—N4	123.1 (3)
C5—N1—H1A	120 (4)	C17—N3—H3A	117 (3)
N2—N1—H1A	115 (4)	N4—N3—H3A	120 (3)
C6—N2—N1	115.9 (3)	C18—N4—N3	116.5 (3)
C2—C1—S1	113.6 (4)	C14—C13—S2	113.8 (4)
C2—C1—H1	123.2	C14—C13—H13	123.1
S1—C1—H1	123.2	S2—C13—H13	123.1
C1—C2—C3	116.2 (5)	C13—C14—C15	113.3 (5)
C1—C2—H2B	121.9	C13—C14—H14A	123.3
C3—C2—H2B	121.9	C15—C14—H14A	123.3
C4—C3—C2	105.8 (4)	C16—C15—C14	109.8 (4)
C4—C3—H3	127.1	C16—C15—H15	125.1
C2—C3—H3	127.1	C14—C15—H15	125.1
C3—C4—C5	120.0 (4)	C15—C16—C17	121.2 (4)
C3—C4—S1	113.4 (3)	C15—C16—S2	111.8 (3)
C5—C4—S1	126.6 (4)	C17—C16—S2	126.9 (4)
O1—C5—N1	119.6 (3)	O2—C17—N3	119.6 (4)
O1—C5—C4	119.5 (4)	O2—C17—C16	119.4 (4)
N1—C5—C4	120.9 (4)	N3—C17—C16	121.1 (4)
N2—C6—C7	120.4 (3)	N4—C18—C19	121.0 (3)
N2—C6—H6A	119.8	N4—C18—H18A	119.5
C7—C6—H6A	119.8	C19—C18—H18A	119.5
C8—C7—C12	116.7 (4)	C20—C19—C24	116.6 (4)
C8—C7—C6	122.0 (3)	C20—C19—C18	122.3 (3)
C12—C7—C6	121.2 (4)	C24—C19—C18	121.1 (4)
C7—C8—C9	122.5 (4)	C19—C20—C21	121.5 (4)
C7—C8—Cl1	120.5 (3)	C19—C20—Cl2	120.8 (3)
C9—C8—Cl1	117.0 (3)	C21—C20—Cl2	117.6 (3)
C10—C9—C8	118.3 (4)	C22—C21—C20	120.1 (4)
C10—C9—H9A	120.8	C22—C21—H21A	119.9
C8—C9—H9A	120.8	C20—C21—H21A	119.9
C11—C10—C9	121.2 (4)	C21—C22—C23	118.7 (4)
C11—C10—H10A	119.4	C21—C22—H22A	120.6
C9—C10—H10A	119.4	C23—C22—H22A	120.6
C10—C11—C12	120.4 (4)	C24—C23—C22	120.8 (4)
C10—C11—H11A	119.8	C24—C23—H23A	119.6
C12—C11—H11A	119.8	C22—C23—H23A	119.6
C11—C12—C7	120.9 (4)	C23—C24—C19	122.3 (4)
C11—C12—H12A	119.6	C23—C24—H24A	118.9
C7—C12—H12A	119.6	C19—C24—H24A	118.9
			
C5—N1—N2—C6	−178.9 (4)	C17—N3—N4—C18	179.7 (4)
C4—S1—C1—C2	1.1 (4)	C16—S2—C13—C14	0.5 (5)
S1—C1—C2—C3	−0.2 (6)	S2—C13—C14—C15	0.3 (7)
C1—C2—C3—C4	−1.1 (6)	C13—C14—C15—C16	−1.1 (7)
C2—C3—C4—C5	−176.0 (4)	C14—C15—C16—C17	−176.5 (4)
C2—C3—C4—S1	1.9 (5)	C14—C15—C16—S2	1.5 (5)
C1—S1—C4—C3	−1.8 (3)	C13—S2—C16—C15	−1.2 (4)
C1—S1—C4—C5	176.0 (4)	C13—S2—C16—C17	176.7 (4)
N2—N1—C5—O1	−179.1 (3)	N4—N3—C17—O2	−179.6 (3)
N2—N1—C5—C4	0.7 (6)	N4—N3—C17—C16	1.6 (6)
C3—C4—C5—O1	5.1 (6)	C15—C16—C17—O2	8.7 (6)
S1—C4—C5—O1	−172.5 (3)	S2—C16—C17—O2	−168.9 (3)
C3—C4—C5—N1	−174.7 (4)	C15—C16—C17—N3	−172.5 (4)
S1—C4—C5—N1	7.6 (6)	S2—C16—C17—N3	9.9 (6)
N1—N2—C6—C7	177.5 (3)	N3—N4—C18—C19	178.0 (3)
N2—C6—C7—C8	−179.1 (3)	N4—C18—C19—C20	−179.3 (3)
N2—C6—C7—C12	−0.2 (6)	N4—C18—C19—C24	−0.1 (5)
C12—C7—C8—C9	−1.3 (6)	C24—C19—C20—C21	−0.6 (5)
C6—C7—C8—C9	177.6 (4)	C18—C19—C20—C21	178.7 (3)
C12—C7—C8—Cl1	179.4 (3)	C24—C19—C20—Cl2	−177.9 (3)
C6—C7—C8—Cl1	−1.7 (5)	C18—C19—C20—Cl2	1.3 (5)
C7—C8—C9—C10	0.5 (6)	C19—C20—C21—C22	0.0 (6)
Cl1—C8—C9—C10	179.9 (4)	Cl2—C20—C21—C22	177.4 (3)
C8—C9—C10—C11	−0.2 (7)	C20—C21—C22—C23	0.8 (6)
C9—C10—C11—C12	0.7 (7)	C21—C22—C23—C24	−1.0 (7)
C10—C11—C12—C7	−1.5 (6)	C22—C23—C24—C19	0.3 (6)
C8—C7—C12—C11	1.7 (6)	C20—C19—C24—C23	0.5 (6)
C6—C7—C12—C11	−177.2 (4)	C18—C19—C24—C23	−178.8 (4)

### Hydrogen-bond geometry (Å, º)

**Table:**

*D*—H···*A*	*D*—H	H···*A*	*D*···*A*	*D*—H···*A*
N3—H3*A*···O1^i^	0.84 (4)	2.02 (5)	2.856 (4)	175 (4)
N1—H1*A*···O2^ii^	0.77 (4)	2.09 (4)	2.845 (5)	166 (5)
C10—H10*A*···Cl2^iii^	0.93	2.93	3.758 (5)	149

Symmetry codes: (i) *x*+1/2, −*y*−1/2, *z*; (ii) *x*−1/2, −*y*−1/2, *z*; (iii) −*x*+1, −*y*+2, *z*+1/2.
